# The expanding role of 16s ribosomal RNA PCR in the management of patients with infective endocarditis undergoing cardiac surgery

**DOI:** 10.3389/fcvm.2024.1504197

**Published:** 2024-12-18

**Authors:** Natalia Pavone, Federico Cammertoni, Maria Calabrese, Piergiorgio Bruno, Giancarlo Scoppettuolo, Antonella Lombardo, Francesca Giovannenze, Eleonora Taddei, Barbara Fiori, Tiziana D’Inzeo, Gessica Cutrone, Giulia Iannaccone, Niccolò Del Zanna, Massimo Massetti

**Affiliations:** ^1^ Department of Cardiovascular Medicine, Fondazione Policlinico Universitario A. Gemelli IRCCS, Rome, Italy; ^2^ Dipartimento di Scienze di Laboratorio e Infettivologiche, Fondazione Policlinico Universitario A. Gemelli IRCCS, Rome, Italy; ^3^ Department of Cardiovascular and Pulmonary Sciences, Catholic University of the Sacred Heart, Rome, Italy; ^4^ Dipartimento di Scienze di Laboratorio ed Ematologiche, Fondazione Policlinico Universitario A. Gemelli IRCCS, Rome, Italy; ^5^ Faculty of Medicine and Surgery, Catholic University of the Sacred Heart, Rome, Italy

**Keywords:** infective endocarditis, heart valve prosthesis, 16s ribosomal RNA PCR, cardiac surgery, molecular test

## Abstract

**Background:**

Aetiological diagnosis and targeted antibiotic therapy are essential to improve the prognosis of patients with infective endocarditis. Molecular tests on blood have been reported to be effective in identifying the causative organism and are recommended when blood cultures are negative. The role of molecular tests on the surgically excised valve is still unclear and needs further investigation.

**Materials and methods:**

In this prospective, observational, single center study, we enrolled 100 consecutive patients with native or prosthetic valve endocarditis who underwent cardiac surgery between April 2020 and June 2023. Results of preoperative blood cultures, valve culture, 16s ribosomal RNA and histopathologic analysis of surgical samples were collected in a dedicated database.

**Results:**

The mean age of the study population was 60 ± 12.5 years, with a majority of men (73%). Previous cardiac surgery was reported in 31% of patients. Blood culture, valve culture, and 16srRNA were positive in 83%, 47%, and 76% of cases, respectively. The sensitivity of both valve culture and 16srRNA decreased significantly with prolonged preoperative antibiotic therapy. Of note, 16srRNA was the only positive result in 7% of cases, allowing aetiological diagnosis. In 33% of patients, the valve culture test was negative while the molecular test was positive. In these cases, histopathological analysis showed acute inflammation in most cases. In 10%, the molecular test helped in resolving discrepancies between the results of blood and valve cultures.

**Conclusions:**

The molecular test showed significantly higher diagnostic sensitivity than valve culture and maintained this efficacy even after 28 days of preoperative antibiotic therapy. In addition to identifying the pathogen in 7% of cases with negative culture results, the molecular test demonstrated utility in other crucial situations. When valve cultures were negative, combining molecular testing and histopathological analysis they allowed the identification of patients who could benefit from prolonged antibiotic therapy. In addition, molecular testing guided the choice of antibiotic treatment when there was a discrepancy between blood culture and valve culture results. Based on these findings, molecular testing should be considered in all patients with infective endocarditis undergoing cardiac surgery.

**Clinical Trial Registration**: ClinicalTrials.gov, identifier (NCT05791357).

## 1 Introduction

The prevalence of infective endocarditis (IE) has doubled over the last 20 years and is now 14 cases per 100,000 people/year thus becoming an alarming public health problem (1, 2). Early aetiological diagnosis and start of targeted antibiotic therapy are crucial to improve prognosis (3). In this sense, blood cultures are the cornerstone of aetiological diagnosis. However, the causative microorganism cannot be identified up to 30% of cases (3) due to previous empirical antibiotic therapy, slow-growing, intracellular, non-cultivable organisms, as well as inaccuracy in blood sample collection, storage or analysis (4, 5). In those circumstances, serological tests followed by specific polymerase chain reaction (PCR) assays on blood should be considered (3, 6, 7).

Approximately half of these patients require surgery (3, 8–12). However, excised tissue culture may be impaired by low sensitivity caused by prolonged preoperative antibiotic therapy (13). Beginning in the 90s, amplification and sequencing of ribosomal RNA genes have been used to identify causative microorganisms (14). Since then, evidence has accumulated to support the use of molecular tests as an alternative, rapid, culture-independent method to identify pathogens in blood culture-negative IE (BCN-IE) or to confirm them in blood culture positive IE (BCP-IE) cases (15). To date, latest guidelines recommend the use of PCR on blood and valve tissue in BCN-IE to identify the aetiological agent (3).

However, whether PCR should be reserved for selected patients or whether it can be useful in multiple contexts is still unclear and it needs further investigation.

The aims of this prospective, observational study are (1) to investigate the potential role of molecular analysis in patients with BCP-IE and (2) to test the performance of PCR in a contemporary cohort of patients who underwent cardiac surgery.

## 2 Materials and methods

Since the launch of a dedicated clinical pathway for patients with IE in our tertiary care hospital, we have included 16srRNA analysis of the excised valve into our practice. Indeed, it is routinely performed for all patients undergoing cardiac surgery for IE, regardless of whether an aetiological diagnosis has been made previously. Every clinical case is multidisciplinary discussed by the local Endocarditis Team with regard to: diagnosis, antibiotic therapy type and duration, instrumental evaluation type and timing, surgical indication and timing, complications management, follow-up plan. With the aim of optimizing treatment and improving prognosis, this multidisciplinary approach ensures a comprehensive and personalized management of patients with IE.

### 2.1 Patients

From April 2020 to June 2023, all adult patients diagnosed with native or prosthetic, definite or suspected IE according to the modified Duke's criteria and identified as candidates for surgery were prospectively evaluated. Exclusion criteria were: (a) patients in whom IE was only identified during surgery, (b) patients with infection of cardiac implantable electronic devices (CIEDs), (c) patients with transcatheter valve prostheses IE. Patients unable to express their informed consent were also excluded by this research protocol. The study was approved by the local ethics committee (ID: 3451) prior to patient enrolment. All participants provided written informed consent after receiving both verbal and written information.

The study protocol was registered on https://clinicaltrials.gov with the following ID NCT05791357.

### 2.2 Data collection

Demographic and clinical information, laboratory and instrumental tests, results of blood cultures, type and duration of antibiotic therapy, surgery data and postoperative outcomes were collected in a dedicated database. During surgery, the excised material was splitted into two parts: one for histological analysis (stored in formaldehyde), one for microbiological analysis, including culture and molecular testing (stored in Ringer's lactate solution). Mechanical prostheses were only used for microbiological analysis.

### 2.3 Microbiological analysis

After vortexing the primary container, the sample was subjected to sonication (BactoSonic, Bandelin, Berlin, Germany), followed by a second vortexing step and then centrifugation for 15 min at 3,500 relative centrifugal force. Then, the concentrate was divided: one part was seeded on agar media (agar blood and agar chocolate) using the spread plate method and then incubated at 35 °C in a 5% CO_2_ atmosphere. Schaedler's plates, Columbia agar and thioglycollate broth were incubated anaerobically at 35 °C.

In addition, 200 µl of the concentrate was transferred to 2 ml Eppendorf tubes and subjected to broad-range bacterial PCR, with amplification primers targeting the bacterial 16S rRNA gene and organism-specific primers for *Tropheryma whipplei*, *Coxiella burnetii* and *Bartonella* species. Following amplification, bacterial identification was determined by sequencing amplified DNA followed by comparison of the sequence to established databases (Supplementary Figure S1).

### 2.4 Statistical analysis

Continuous variables were reported as mean ± standard deviation if normally distributed or as median (interquartile range), otherwise. The Kolmogorov-Smirnov test was used to determine the type of distribution of continuous variables. Categorical variables were reported as absolute number and percentages. Continuous variables were compared using Student's *t*-test or Mann–Whitney *U*-test, as appropriate. Categorical variables were compared using the chi^2^ test or Fisher's exact test, as appropriate. A *p* value less than 0.05 deemed statistically significant. Binary logistic regression analysis was performed to predict the probability of positive 16srRNA and valve culture according to the duration of preoperative antibiotic therapy. SPSS version 23 (SPSS Inc) was used for statistical analysis.

## 3 Results

### 3.1 Study population

Overall, 100 consecutive patients were included in the study. Seventy (70%) and thirty (30%) patients met the Duke's criteria for definite and possible diagnosis of endocarditis, respectively. As shown in Table 1, mean age was 60.0 ± 12.5 years and patients were mostly men (73%). Six patients (6%) were habitual intravenous drug abusers. Of note, 31% of cases had undergone previous cardiac surgery. Endocarditis affected the aortic, mitral or tricuspid valve in 45%, 32% and 6% of patients, respectively (Supplementary Figure S2). Concomitant aortic and mitral valve IE occurred in 15% of cases. A clear vegetation was observed in 79% of cases, while abscess (23%), leaflet perforation (19%) and pseudoaneurysms (11%) were less common. Septic embolization was detected in 62% of patients, with 20% and 7% presenting in septic or cardiogenic shock, respectively. The risk assessment revealed that patients with native valve endocarditis had significantly lower surgical risk compared to those with prosthetic valve endocarditis [EuroScore II: 3.3 (1.9–5.8) vs. 9.1 (4.5–16.7), *p* < 0.01]. At the time of surgery, patients had been on antibiotic therapy for a median duration of 13 days (7–33). Preoperative procalcitonin and C-reactive protein were 0.3 ng/ml (0.1–0.5) and 48.4 mg/L (19.4–107.2), respectively (Supplementary Table S1).

**Table 1:** Baseline characteristics.

	Entire study population (*n* = 100)
Male	73 (73)
Age, years	60.0 ± 12.5
Age > 80 years	3 (3)
BMI, Kg/m^2^	25.7 ± 4.5
BMI > 30 Kg/m^2^	14 (14)
BMI < 18 Kg/m^2^	2 (2)
Active smoker	23 (23)
Hypertension	58 (58)
Diabetes	16 (16)
Dyslipidemia	22 (22)
NYHA class	
NYHA I	18 (18)
NYHA II	31 (31)
NYHA III	34 (34)
NYHA IV	17 (17)
COPD	3 (3)
PVD	7 (7)
AF	30 (30)
Creatinine Clearance, ml/min	79.5 ± 31.3
Renal function	
Normal	46 (46)
Moderately impaired	37 (37)
Severely impaired	17 (17)
Previous PMK^a^	8 (8)
Previous Cardiac Surgery	31 (31)
Valve surgery with tissue valve	20 (64.5)
Valve surgery with mechanical valve	8 (25.8)
Other	3 (9.7)
IVDU	6 (6)
EuroSCORE II	
Overall	4.2 (2.1–10.7)
Native valve endocarditis	3.3 (1.9–5.8)
Prosthetic valve endocarditis	9.1 (4.5–16.7)

Categorical data were presented as *n* (%). Continuous data were summarized as mean ± standard deviation or median (1st–3rd quartile); AF, atrial fibrillation; BMI, body mass index; COPD, chronic obstructive pulmonary disease; IVDU, intravenous drug user; NYHA, New York Heart Association; PMK, pacemaker; PVD, peripheral vascular disease.

^a^ These patients had either native or prosthetic valve endocarditis without PMK's leads involvement.

### 3.2 Postoperative outcomes

Seven patients (7%) died within 30 days after surgery, mainly due to septic shock (57%) (Table 2). All these patients have had positive blood cultures and were on targeted antibiotic therapy. Of note, 5 out of 7 patients had received targeted therapy for less than 2 weeks. Postoperative intensive care unit (ICU) and hospital length of stay were 3 (2–5) and 34 days (19–51), respectively. For 80% of patients, red blood cells transfusions were needed. Similarly, fresh frozen plasma (46%) and platelets (21%) transfusions were common. Postoperative bleeding requiring surgical revision was the most common complication occurring in 17% of cases.

**Table 2:** Mortality and postoperative outcomes.

	Entire study population (*n* = 100)
Mechanical ventilation, hours	17 (12–26)
Inotropes	70 (70)
ECMO	1 (1)
Postoperative stroke	5 (5)
Bleeding requiring surgery	17 (17)
Deep wound complications	3 (3)
Postoperative AF	29 (29)
PMK	9 (9)
AKI requiring dialysis	9 (9)
Transfusions	
RBC	80 (80)
PLT	21 (21)
FFP	46 (46)
ICU stay, days	3 (2–5)
Hospital stay, days	34 (19–51)
30-day mortality	7 (7)
Septic shock	4 (57)
Cardiogenic shock	2 (29)
Acute intestinal ischemia	1 (14)

Categorical data were presented as *n* (%). Continuous data were summarized as median (1st–3rd quartile).

AF, atrial fibrillation; AKI, acute kidney injury; ECMO, extracorporeal membrane oxygenation; FFP, fresh frozen plasma; ICU, intensive care unit; PMK, pacemaker; PLT, platelets; RBC, red blood cells.

### 3.3 Microbiological tests

#### 3.3.1 Tests sensitivity

**Figure 1:**
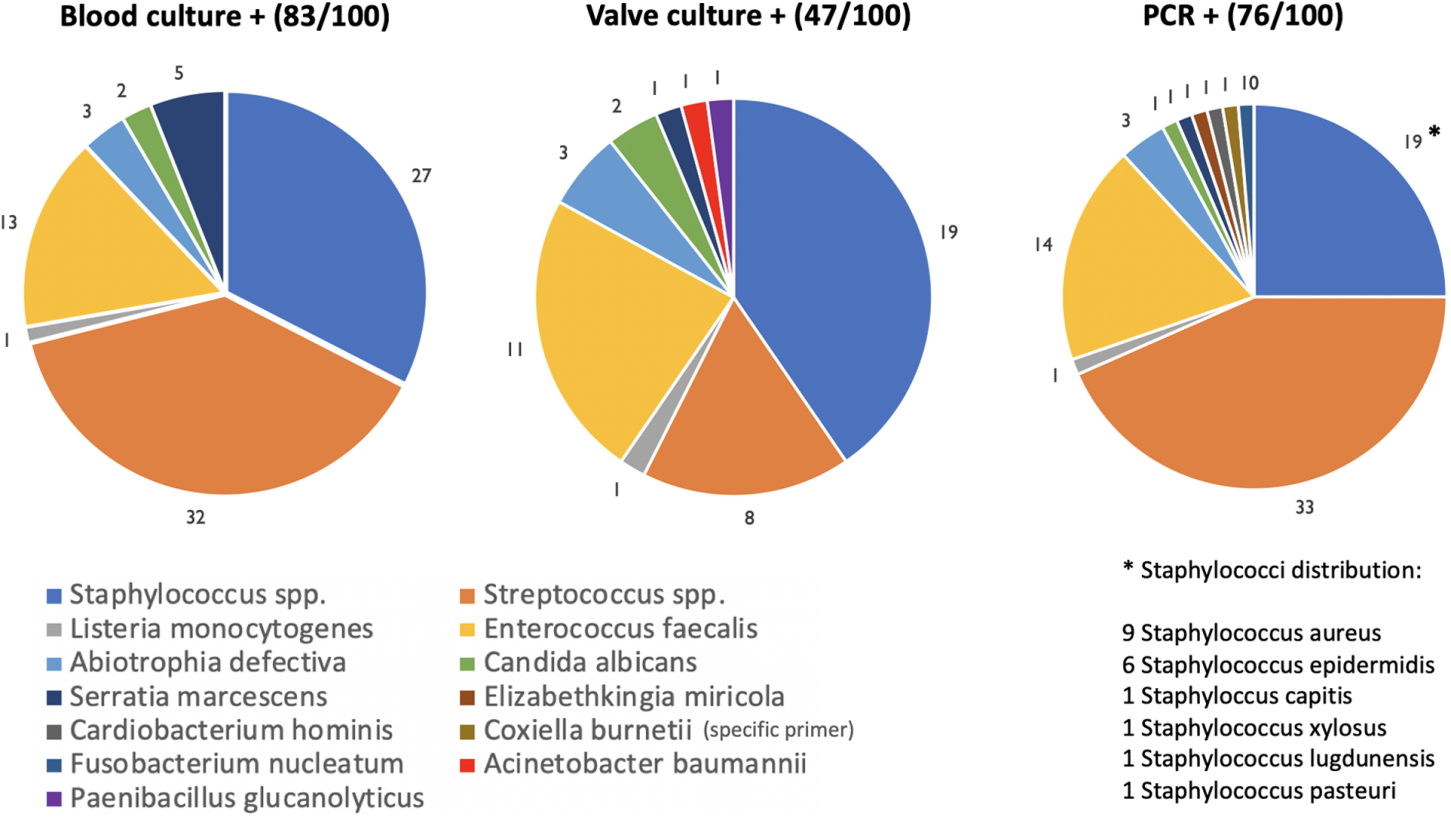
Pie charts showing results of blood cultures, valve cultures and 16srRNA PCR.

Eighty-three patients (83%) had positive blood cultures and were on targeted antibiotic therapy at the time of surgery. The remaining 17% were classified as BCN-IE. For these patients, specific serological tests were performed but were only available after surgery. Valve culture and 16srRNA were performed in all patients and showed sensitivities of 47% and 76%, respectively (Figure 1).

#### 3.3.2 The impact of preoperative antibiotic therapy on microbiological tests sensitivity

When stratified by the duration of preoperative antibiotic therapy, both valve culture and 16srRNA sensitivities decreased with increasing duration of antibiotic treatment (Table 3). However, while valve culture sensitivity decreased quickly, 16srRNA sensitivity showed a slower reduction: at 28 days of antibiotic therapy, sensitivity was 60% vs. 20% (*p* < 0.01) for PCR vs. valve culture, respectively.

**Table 3:** Influence of pre-operative antibiotic therapy on valve culture and PCR results.

	*n* = 100	Valve culture (+)	PCR (+)	*p*
Less than 14 days before surgery	50 (50)	38 (76)	43 (86)	0.31
Within 14 and 28 days before surgery	18 (18)	2 (11)	14 (78)	<0.01
More than 28 days before surgery	30 (30)	6 (20)	18 (60)	<0.01
Uncertain	2 (2)	1 (1)	1 (1)	
Pre-operative antibiotic therapy duration (days)	OR: 0.94 (CI 95%: 0.91–0.97) *p* < 0.001		OR: 0.97 (CI 95%: 0.95–0.99) *p* = 0.01	
Pre-operative antibiotic therapy < 14 days	OR: 14.23 (CI 95:% 5.30–38.21) *P* < 0.001		OR: 3.07 (CI 95%: 1.13–8.34) *p* = 0.03	

Data are summarized as number (%).

CI 95%, 95% confidence interval; PCR, polymerase chain reaction; OR, odds ratio.

#### 3.3.3 Microbiological tests results comparison

**Figure 2:**
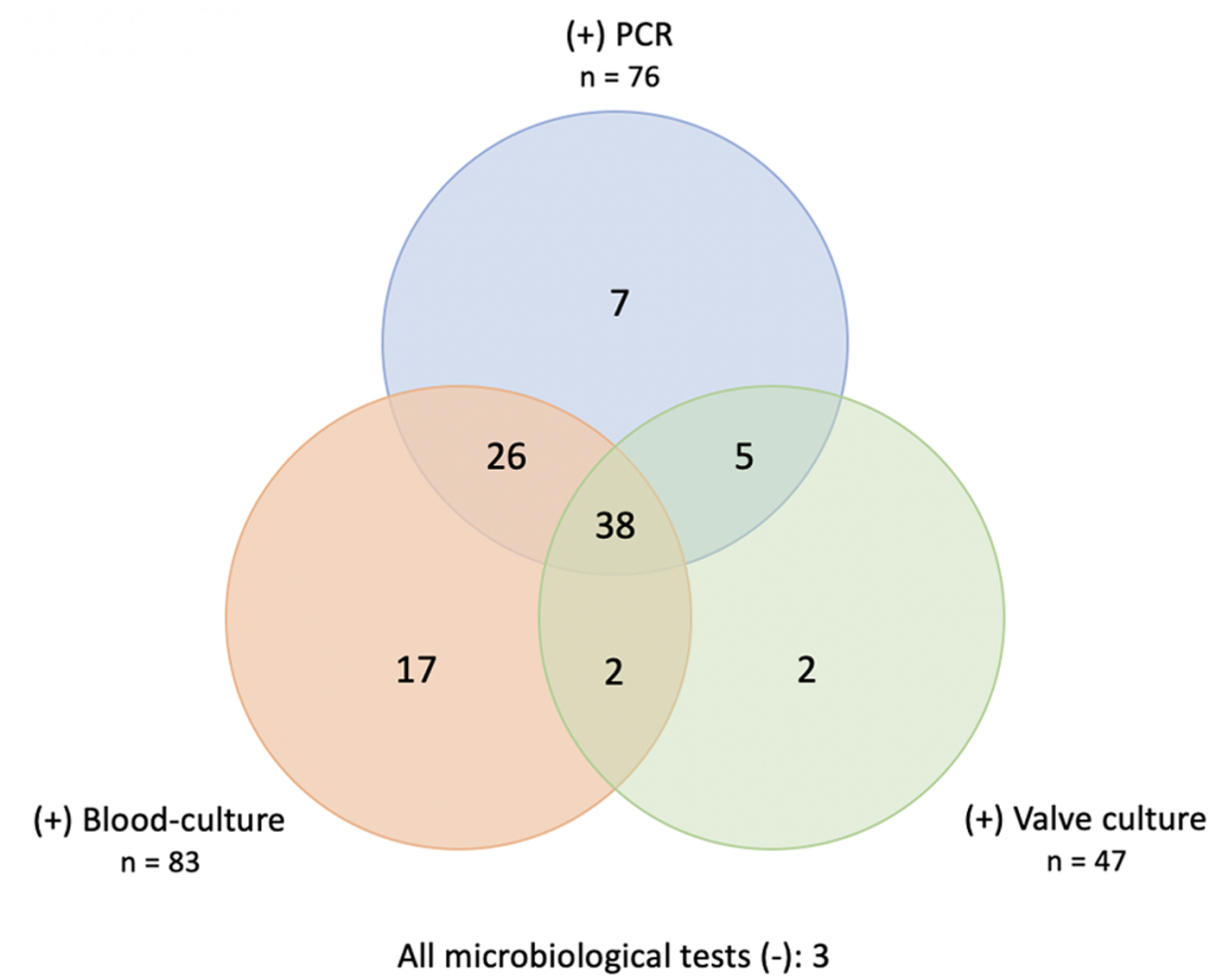
Venn-diagram matching positive results of blood cultures, valve cultures and 16srRNA PCR.

As shown in Figure 1, the most common causative organisms were streptococci (33%), staphylococci (19%) and enterococci (14%). The Venn diagram (Figure 2) matches the results of the three microbiological assays. For 3 patients (3%) all tests were negative. Nevertheless, histopathological examination revealed signs of acute inflammation and bacteria. So, these patients were classified as BCN-IE and treated accordingly. In 7 patients (7%) 16srRNA was the only positive test allowing an aetiological diagnosis. In these cases, streptococci were the most common microorganisms identified (4/7: 2 *Streptococcus agalactiae*, 1 *Streptococcus oralis*, 1 *Streptococcus tigurinus*); in the remaining 3 cases/7: *Enterococcus faecalis*, *Coxiella burnetii* and *Fusobacterium nucleatum*. Interestingly, 33 patients (33%) with negative valve culture had a positive 16srRNA. Of them, 26 out of 33 had preoperative positive blood cultures. In addition, 4 out of 100 patients had a negative molecular test despite a positive valve culture. Specifically, the microorganisms isolated were 2 *Streptococcus gordonii*, 1 *Candida albicans* and 1 *Enterococcus faecalis*.

#### 3.3.4 Concordance analysis

Forty out of 100 patients had positive both blood and valve cultures. Of these, 36 (90%) showed concordant species. In the remaining 10%, 16srRNA was useful to clarify aetiological diagnosis. Supplementary Figure S3 reports these four cases and describes how each patient was managed.

Of the 76 out of 100 patients with a positive valve 16srRNA, 64 also had a positive blood culture. In 54 out of 64 patients (84%), concordant bacterial species were identified.

Finally, 43 out of 100 patients had both positive valve PCR and valve culture, with 39 (90%) showing concordant microorganisms.

### 3.4 Histological examination

Histological analysis of the excised specimen was available for 85 patients (85%). Among the remaining 15 patients, 6 had a mechanical valve and in 9 histological examinations could not be performed due to either insufficient or inadequate samples. Mostly (73 patients, 86%), signs of acute inflammation were found, with bacterial colonies noticed in 39 out of 73 cases. Chronic inflammation occurred in 9 patients (10%), while no inflammation was detected in 3 patients (4%).

Histology reports were available for 64 out of 76 patients with positive 16srRNA and more frequently showed acute inflammation (89%). Finally, histology was performed in 30 of 33 patients with a negative valve culture but positive 16srRNA. Of these, 26 out of 30 patients (87%) showed clear signs of acute inflammation, with bacterial identification in 10 out of 26 cases (38%). The remaining 4 out of 30 patients showed evidence of chronic inflammation.

## 4 Discussion

Infective endocarditis is becoming a major clinical challenge (16–18). Early identification of the causative pathogen and prompt initiation of targeted antibiotic therapy are essential to improve patient prognosis. However, even when correctly carried out, blood cultures could result negative in 2%–30% of cases (3, 17) due to previous antibiotic treatment or to slow-growing, intracellular, fastidious pathogens (4, 5). Surgically-excised native or prosthetic valve culture could help in microbiological diagnosis. However, its accuracy is compromised by either preoperative antibiotic therapy (false negative), and the risk of contamination during specimen collection and processing (false positive) (19–21). Over the past two decades, there has been considerable evidence that molecular tests could be a valuable resource in these cases. These techniques offer rapid and growth-independent results (13–15). The 2023 guidelines for the management of infective endocarditis recommended the use of 16S and 18S rRNA sequencing from tissue samples when blood cultures are negative (3). It remains to be determined whether molecular tests should be limited to BCN-IE, or whether they could offer benefits in other clinical contexts.

The first finding of this study was that the sensitivity of microbiological tests was in line with other reports. Specifically, blood cultures, valve cultures and 16srRNA had a sensitivity of 83%, 47% and 76%, respectively. In a retrospective analysis of 146 patients, Armstrong et al. reported a sensitivity of 68% for 16S rDNA PCR (12). More recently, Mularoni and colleagues (13) documented higher PCR sensitivity (88%) in a cohort of 137 patients, comparable to what previously reported by Peeters et al. (87%) and Shrestha et al. (90%) (21, 22).

In a retrospective, single-center study involving 87 patients with IE, Haalavaara and coauthors (9) showed that the overall sensitivity of molecular analysis was 74%. However, it was significantly influenced by the duration of preoperative antibiotic therapy, ranging from 91% for patients on antibiotics for less than 2 weeks to 53% for patients on antibiotics for more than 2 weeks. Similarly, we found that the duration of preoperative antibiotic therapy greatly impacted the sensitivity of microbiological tests. Both valve culture and molecular testing showed a significant decrease of sensitivity with prolonged preoperative antibiotic therapy. Nevertheless, while the molecular test maintained a sensitivity of 60% even after 28 days or more of antibiotic therapy, valve culture had a residual sensitivity of 20%. Similarly, Vollmer et al. reported that although PCR could identify bacterial DNA mainly within the first 20 days after antibiotic therapy initiation, it was still positive in a considerable percentage of cases thereafter (15). Previously, Kotilainen and colleagues had found that the aetiological agent of IE was identified by PCR up to 58 days after the start of antibiotic therapy, whereas the culture tests were negative after only a few days (23).

At this point, it should be acknowledged that a positive molecular test does not necessarily indicate the presence of living bacteria nor an active infection. Indeed, it has been documented that the bacterial genome can persist on the valve even after a complete course of antibiotic therapy (12, 23, 24).

In this sense, histopathological findings are essential to properly handle the result of molecular tests. Among the 64 out of 100 patients with a positive PCR who also had histopathology results, the majority (89%) still showed signs of active inflammation, but in a non-negligible 11% of cases the inflammation was chronic or absent.

Beyond sensitivity, when both blood and valve cultures were negative 16srRNA proved to be useful the most. In these 7% of patients, it led to aetiological diagnosis and initiation of targeted antibiotic therapy. Similar findings were reported by Armstrong et al. who found 13% of patients with positive PCR results despite negative cultures (12). Also, Peeters et al. documented that 9 out of 127 patients (7%) received an aetiological diagnosis through PCR (21). Finally, Kim and coworkers observed that in 13.6% of patients PCR alone was positive, providing an aetiological diagnosis (25).

Of note, the majority of cases were not caused by difficult-to-grow but microorganisms that were not growing due to early and prolonged antibiotic therapy. In this context, the growth-independence of molecular tests was an unquestionable advantage.

**Figure 3:**
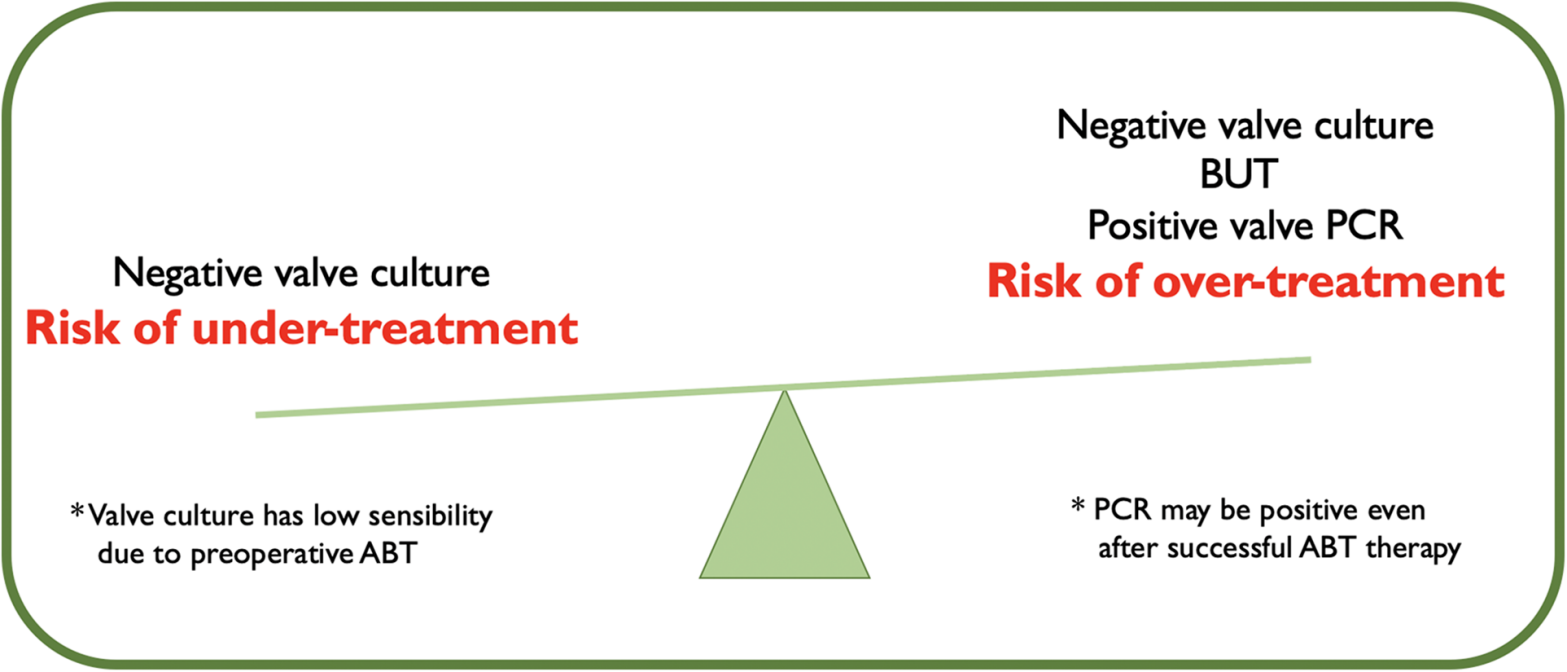
Management of postoperative antibiotic therapy (ABT) according to the result of valve culture and molecular test.

The utility of molecular testing was not limited to BCN-IE. As known, antibiotic therapy duration should be tailored on the result of valve culture, being prolonged in the case of a positive result (23). However, the reduced valve culture sensitivity carries the risk of missing a significant number of patients requiring adjustment in antibiotic duration, potentially leading to undertreatment (Figure 3). In our study, 33% of patients had a negative valve culture but a positive 16srRNA. The management of these patients remains controversial: should the valve be considered still infected and the antibiotic therapy prolonged or should the PCR positivity be considered as the persistence of inactive bacterial DNA on a sterilized valve?

In our experience, 86% of patients with positive PCR but negative valve culture had signs of acute inflammation at histopathological analysis. Also, a non-negligible 10 out of 26 patients showed bacterial colonies. All these patients would have gained from an antibiotic therapy time extension.

Molecular analysis played a role in resolving discrepancies between blood and valve culture. In all four cases (10%) of discordance, PCR helped in the process of providing a definite diagnosis. However, this process cannot consider only PCR but it must take into consideration other issues, such as the specific pathogens (Staph. epidermidis and Staph. cohnii may be contaminants) and the quantitative results of cultures (i.e., number of bottles positive). Also, discrepancies have been found between PCR and either blood or valve culture. Handling these situations may be challenging and a case-by-case approach should be considered as the decision to modify antibiotic therapy is multifactorial. So, every discordance between two tests should be interpreted relying on the result of the third, taking into account patient's medical history, potential portals of entry, specific pathogens and the possibility of sample contamination. Not least, the chance of a polymicrobic infection should be suspected.

In all these three scenarios, (7% of patients with an etiological diagnosis by molecular analysis, 33% of patients with a negative valve culture but positive PCR, and 10% of patients with blood vs. valve cultures discordance) molecular tests would have influenced the type and duration of postoperative antibiotic therapy. Reports by Ursenbach et al. and Fida et al. concluded that molecular methods could influence antibiotic therapy in 16% and 22% of cases, respectively (26, 27).

Molecular tests can produce false negative results. In our experience we had a 4% false negative PCR. One occurred in a case of fungal IE and we did not use specific primers. Conversely, Mularoni and coworkers reported a 12% rate of false negative molecular test results in a cohort of patients with active IE (13). Similarly, Peeters et al. found a 12% rate of false negative results in their series (21). Basing on these data, 1 patient out of 10 could have a premature suspension of antibiotic therapy if only PCR would be used. The reasons for these false negatives could be multiple: low bacterial colonization, timing of surgery, sampling errors or mutations in the target region of the primers. Not less important, as infected material may not be uniformly distributed on the sample, it is critical to analyze the correct part of the excised valve. Regarding this aspect, it remains uncertain whether studying multiple fragments could improve test sensitivity.

### 4.1 Study limitations

Firstly, it is a single-center study so that epidemiology of the different aetiological agents is geographically limited and cannot be generalized. Secondly, a control group of patients without IE is lacking and prevented calculation of the positive predictive value, negative predictive value and specificity of the different microbiological tests. Thirdly, our results cannot be extended to endocarditis on intracardiac devices and transcatheter valves. We did not include this latter group because we believed that such patients, eligible for surgery, would have been very few. Since then, patients with transcatheter valve IE who could be considered for surgery is growing and now represent an important population.

Also, it should be noted that molecular testing provides information on the presence or absence of the bacterial genome, but not on the viability of the pathogen and its susceptibility to antibiotics. This latter aspect is of increasing interest and was not investigated in our study.

Then, this was a retrospective analysis of prospectively collected data. At the time of study protocol writing, which was in line with current guidelines, we did not contemplate the possibility to change the duration of antibiotic therapy for those patients who were found to have positive PCR but negative valve culture. Moreover, PCR turnaround time was variable and so it would not have made possible to effectively modify medical therapy. So, we can only speculate on the effective impact of this measure on patients' outcomes. Finally, a cost-benefit analysis is missing, which is particularly relevant given that molecular methods currently have not-negligible costs and are not universally available.

## 5 Conclusion

**Figure 4:**
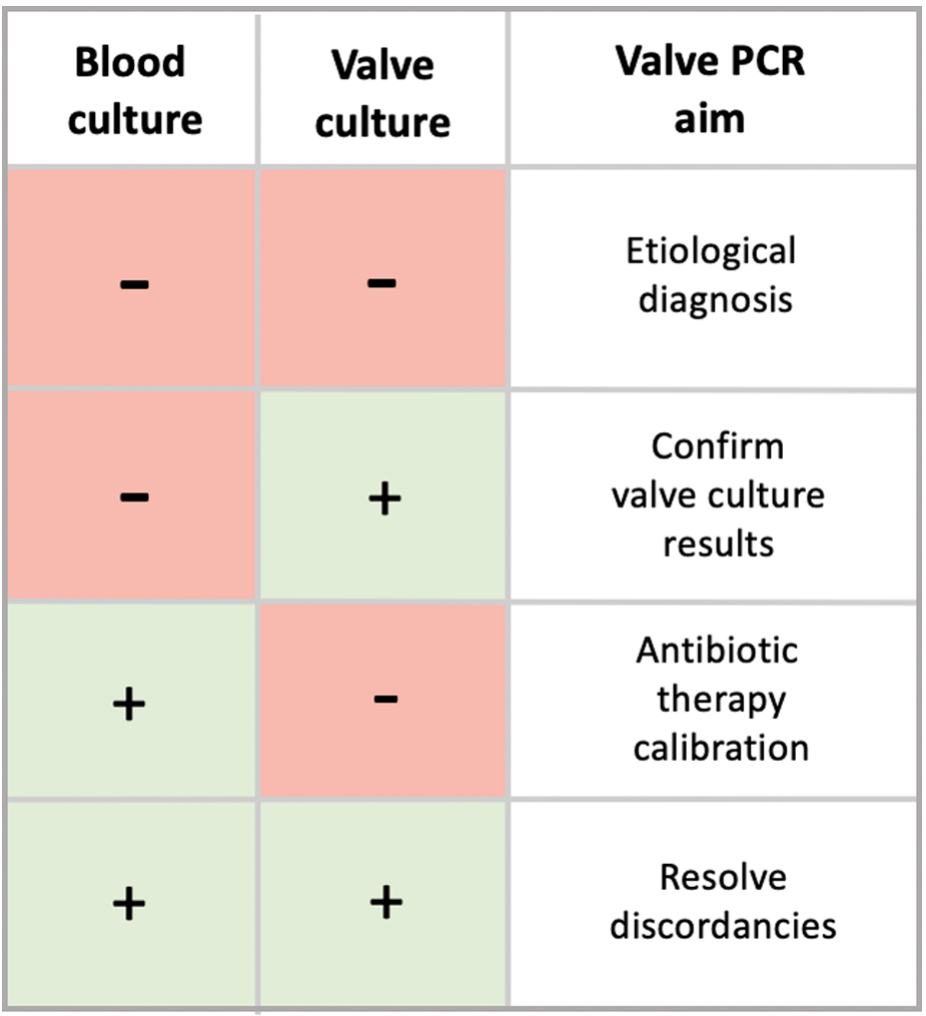
Summary table reporting the role of 16srRNA PCR according to the results of both blood and valve cultures.

**Figure 5:**
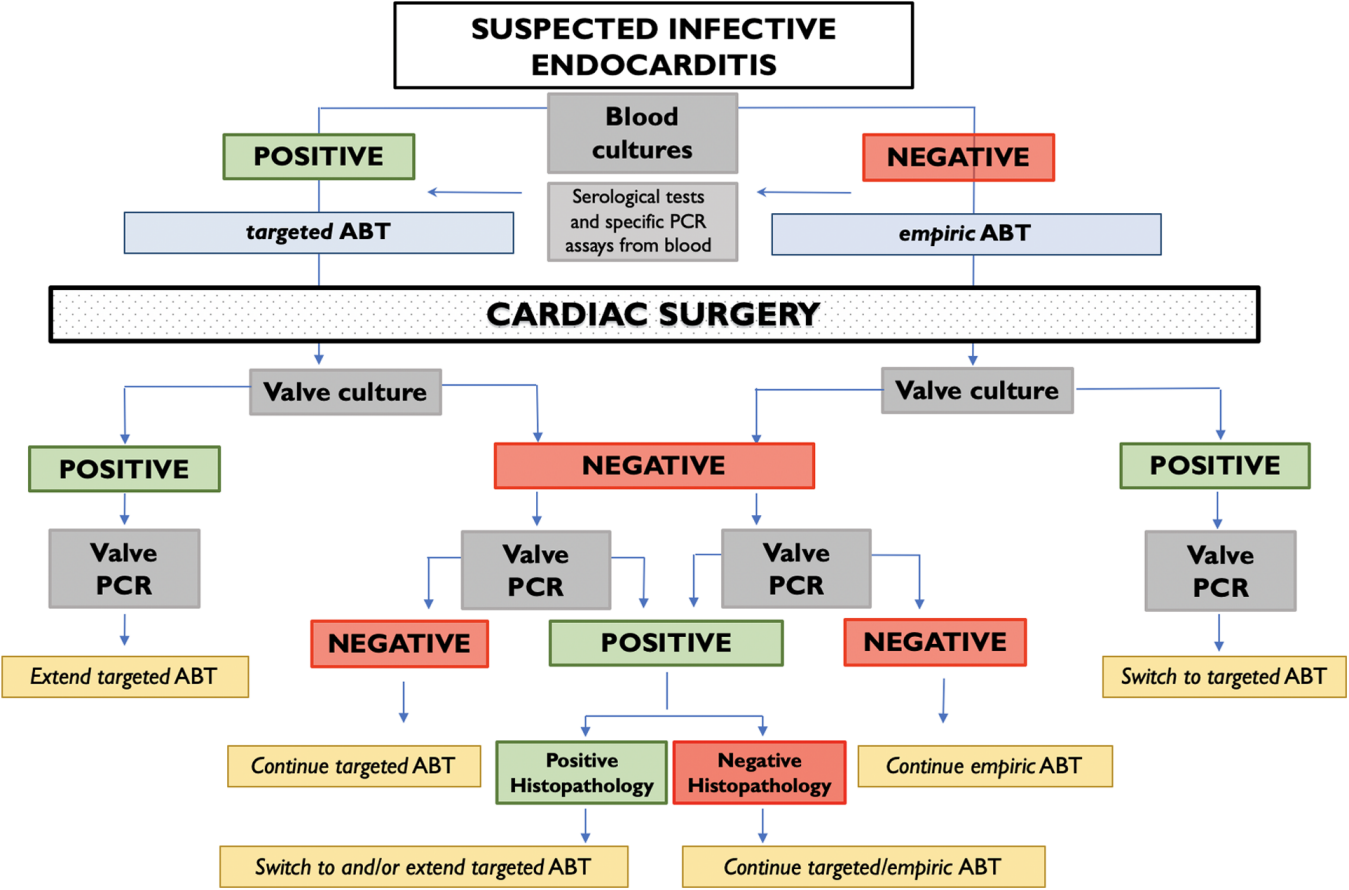
Workflow chart integrating 16srRNA PCR in the management of antibiotic therapy for infective endocarditis for patients undergoing cardiac surgery.

In our experience, 16srRNA has been shown to be useful in many situations (Figure 4). First, in cases of BCN-IE, as it can provide aetiological diagnosis. Secondly, when valve culture is negative, PCR along with histopathology could identify those patients who would benefit from a prolongation of antibiotic therapy. Finally, molecular test could resolve discordances between blood and valve cultures results in a significant number of patients. In conclusion, we believe that molecular analysis provides critical information for the optimal management of patients with IE and should be performed systematically on surgical specimens according to the workflow proposed in Figure 5.

## Data Availability

The raw data supporting the conclusions of this article will be made available by the authors, without undue reservation.

## Supplementary material

Supplementary Figure S1 Flow-chart describing the process of microbiological analysis of surgical samples.

Supplementary Figure S2 In this histogram, patients are gathered according to the valve(s) damaged by infective endocarditis. Prosthetic valves endocarditis are shown (green bars).

Supplementary Figure S3 Discordances between blood and valve cultures occurred in 4 patients out of 40 who had positive both tests (10%). In each case, PCR together with the accurate interpretation of the qualitative and quantitative results of cultures, allowed to obtain an aetiological diagnosis.
