# Supplementary figures and images for: The expanding role of 16s ribosomal RNA PCR in the management of patients with infective endocarditis undergoing cardiac surgery
Source: Front Cardiovasc Med. 2024 Dec 18;11:1504197. doi: 10.3389/fcvm.2024.1504197 (PMC11688630; doi:10.3389/fcvm.2024.1504197)

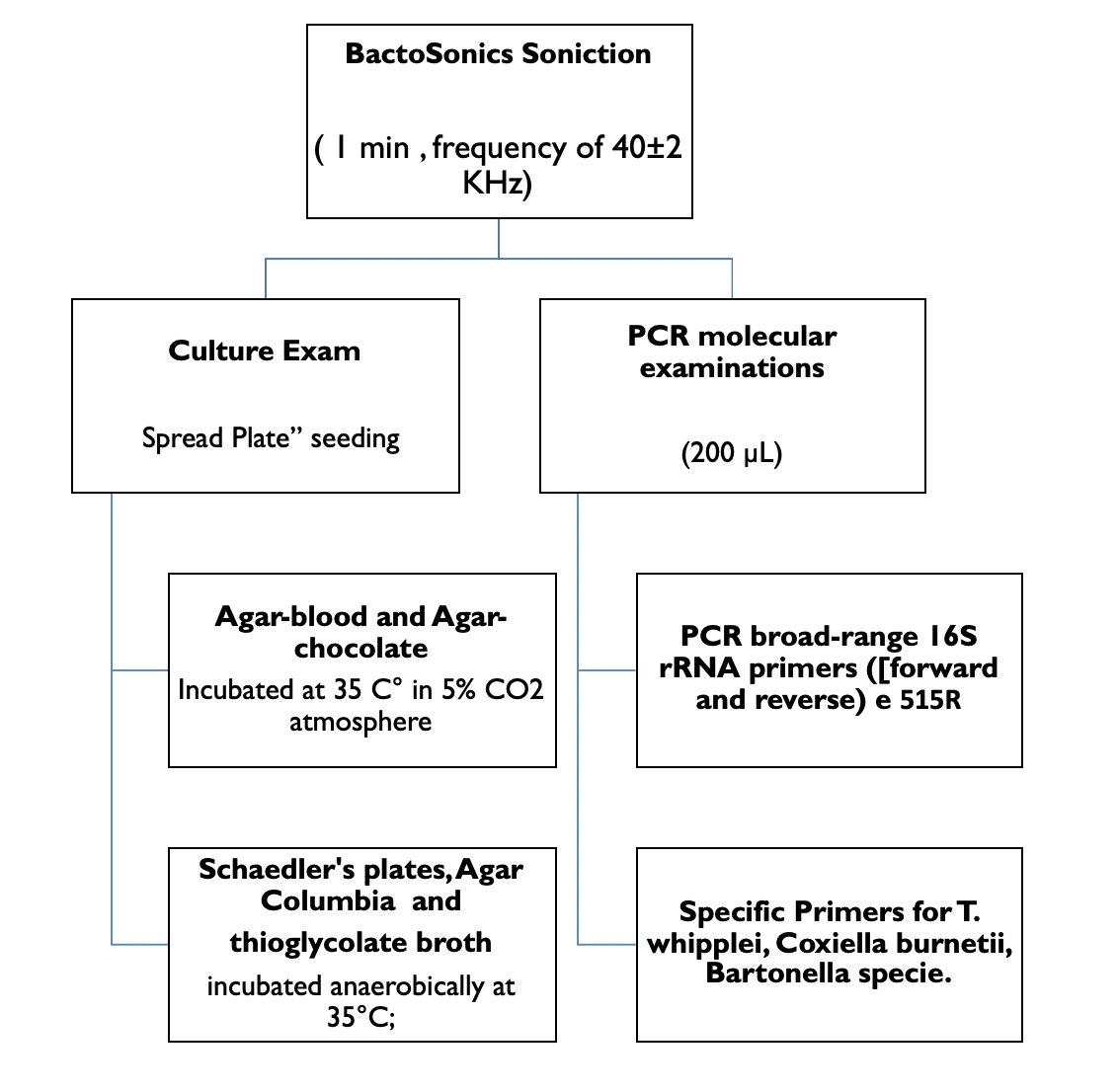

Supplement: Supplementary Figure S1 — Flow-chart describing the process of microbiological analysis of surgical samples. [file Image1.tiff]

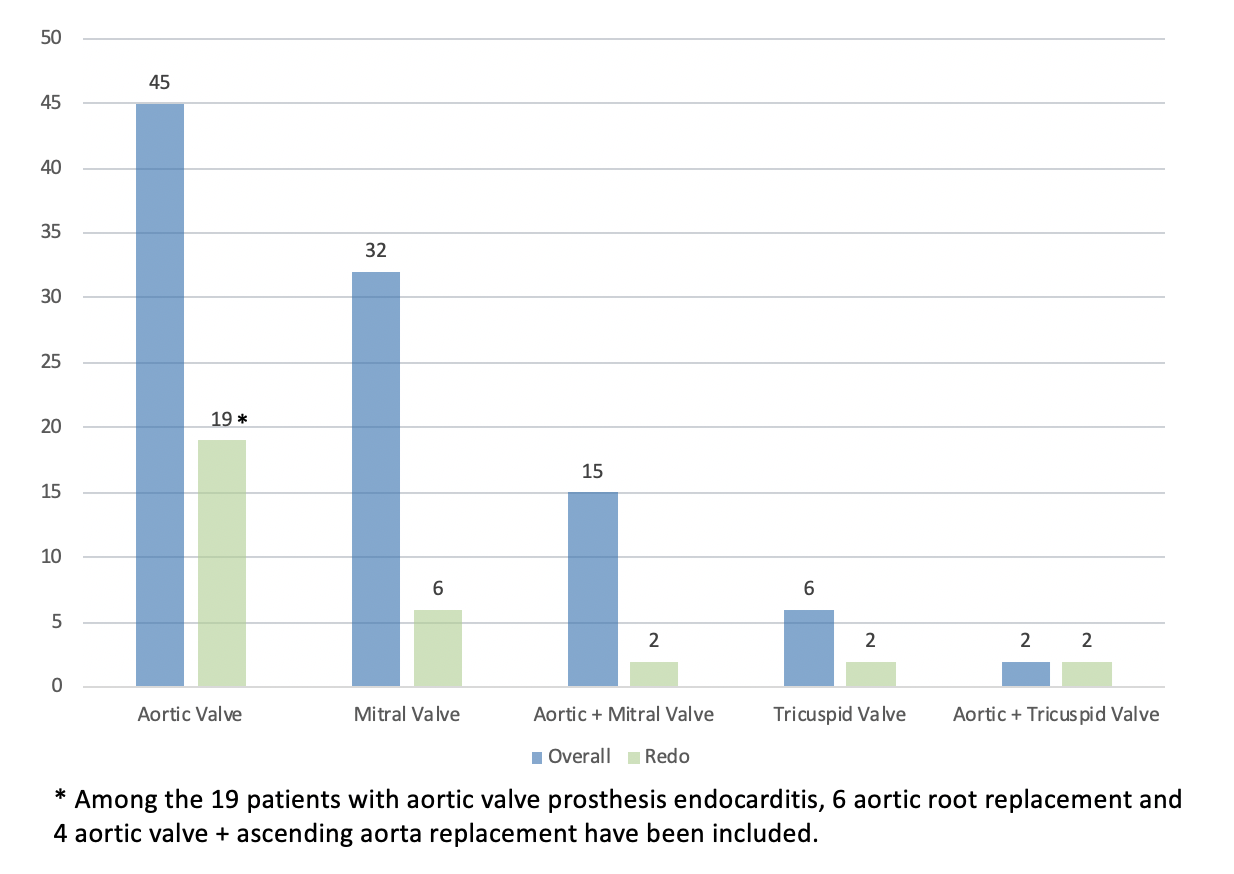

Supplement: Supplementary Figure S2 — In this histogram, patients are gathered according to the valve(s) damaged by infective endocarditis. Prosthetic valves endocarditis are shown (green bars). [file Image2.tiff]

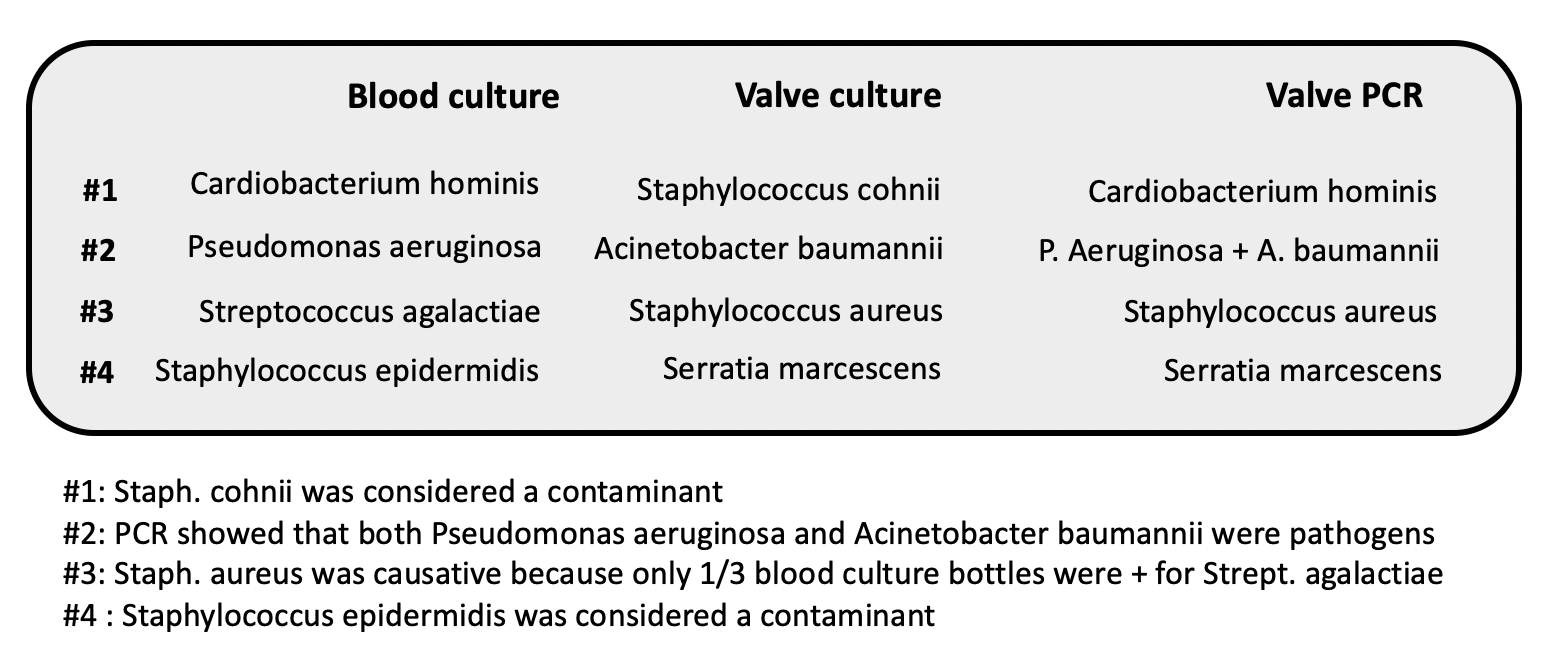

Supplement: Supplementary Figure S3 — Discordances between blood and valve cultures occurred in 4 patients out of 40 who had positive both tests (10%). In each case, PCR together with the accurate interpretation of the qualitative and quantitative results of cultures, allowed to obtain an aetiological diagnosis. [file Image3.tiff]
