# Supplementary material for: The expanding role of 16s ribosomal RNA PCR in the management of patients with infective endocarditis undergoing cardiac surgery
Source: Front Cardiovasc Med. 2024 Dec 18;11:1504197. doi: 10.3389/fcvm.2024.1504197 (PMC11688630; doi:10.3389/fcvm.2024.1504197)
Supplement: Supplementary file 4 [file Table1.docx]

**Supplementary Table S1. Clinical presentation, laboratory and echocardiographic findings**

|  | **Entire Study Population**  **(n = 100)** |
| --- | --- |
| **Endocarditis**  **Modified Duke Criteria**  Definite  Possible | 70 (70)  30 (30) |
| **Septic embolism**  Single  Multiple  Brain  Spleen | 62 (62)  33 (53)  29 (47)  29 (47)  36 (58) |
| **Cardiogenic Shock** | 7 (7) |
| **Septic Shock** | 20 (20) |
| **ET** | 11 (11) |
|  |  |
| **Hemoglobin,** g/dl | 10.3 ± 1.4 |
| **White Blood Cells,** *10^9 / L | 8.6 (6.4 – 11.5) |
| **Platelet Count,** *10^9 / L | 232 (165 – 288) |
| **Procalcitonin,** ng/ml | 0.3 (0.1 – 0.5) |
| **CRP,** mg/L | 48.4 (19.4 – 107.2) |
| **Fever** | 14 (14) |
|  |  |
| **LVEF,** % | 55. 6 ± 10.2 |
| **PAPS,** mmHg | 41.4 ± 14.7 |
| **Type of valve lesion**  Valve perforation  Periannular abscess  Vegetations  Pseudoaneurysm | 19 (19)  23 (23)  79 (79)  11 (11) |

Categorical data were presented as n (%). Continuous data were summarized as mean ± standard deviation or median (1^st^ – 3^rd^ quartile);

CRP = C-Reactive Protein; ET = Endotracheal tube; LVEF = Left Ventricle Ejection Fraction; PAPs = Pulmonary arterial pressure
